# Cancer Treatment in Malawi: A Disease of Palliation

**DOI:** 10.4021/wjon683w

**Published:** 2013-07-15

**Authors:** Claire E Kendig, Jonathan C Samuel, Anna F Tyson, Amal L Khoury, Laura P Boschini, Charles Mabedi, Bruce A Cairns, Carlos Varela, Carol G Shores, Anthony G Charles

**Affiliations:** a Department of Surgery, University of North Carolina, CB 7228, Chapel Hill NC, USA; b NC Jaycee Burn Center, University of North Carolina, CB7600, Chapel Hill NC, USA; c Department of Surgery, Kamuzu Central Hospital, PO Box 149, Lilongwe, Malawi; d Department of Otolaryngology, Head and Neck, University of North Carolina, CB 7070, Chapel Hill NC, USA

**Keywords:** Neoplasms, Africa, Surgery, AIDS, Treatment

## Abstract

**Background:**

Worldwide, new cancer cases will nearly double in the next 20 years while disproportionately affecting low and middle income countries (LMICs). Cancer outcomes in LMICs also remain bleaker than other regions of the world. Despite this, little is known about cancer epidemiology and surgical treatment in LMICs. To address this we sought to describe the characteristics of cancer patients presenting to the Surgery Department at Kamuzu Central Hospital in Lilongwe, Malawi.

**Methods:**

We conducted a retrospective review of adult (18 years or older) surgical oncology services at Kamuzu Central Hospital in Lilongwe, Malawi from 2007 - 2010. Data obtained from the operating theatre logs included patient demographics, indication for operative procedure, procedure performed, and operative procedures (curative, palliative, or staging).

**Results:**

Of all the general surgery procedures performed during this time period (7,076 in total), 16% (406 cases) involved cancer therapy. The mean age of male and female patients in this study population was 52 years and 47 years, respectively. Breast cancer, colorectal cancer, gastric cancer, and melanoma were the most common cancers among women, whereas prostate, colorectal, pancreatic, and, gastric were the most common cancers in men. Although more than 50% of breast cancer operations were performed with curative intent, most procedures were palliative including prostate cancer (98%), colorectal cancer (69%), gastric cancer (71%), and pancreatic cancer (94%). Patients with colorectal, gastric, esophageal, pancreatic, and breast cancer presented at surprisingly young ages.

**Conclusion:**

The paucity of procedures with curative intent and young age at presentation reveals that many Malawians miss opportunities for cure and many potential years of life are lost. Though KCH now has pathology services, a cancer registry and a surgical training program, the focus of surgical care remains palliative. Further research should address other methods of increasing early cancer detection and treatment in such populations.

## Introduction

The increasing burden of cancer-related morbidity and mortality in low to middle income countries (LMICs) has been largely neglected by global public health initiatives [1]. New cancer cases worldwide will increase from 12.7 million in 2008 to 21.4 million in 2030 with 13 million deaths, and disproportionately affect LMICs [2, 3]. Furthermore, cancer outcomes in sub-Saharan Africa lag well behind other regions [4].

The continuing shift in the burden of disease in sub-Saharan Africa away from infectious diseases to non-communicable diseases such as cardiovascular disease and cancer is alarming [5, 6]. As the mortality from HIV/AIDS decreases and the population ages, chronic diseases and cancer will continue to become more prevalent. Despite worse outcomes and an increasing disease burden little is known about the surgical treatment of cancer in sub-Saharan Africa.

To better understand cancer epidemiology in Malawi and guide future efforts to improving surgical capacity and cancer care, we sought to describe the characteristics of cancer patients undergoing surgery at Kamuzu Central Hospital in Lilongwe, Malawi, including demographics, diagnosis, and therapeutic intent.

## Methods

We conducted a retrospective review of all adult (18 years or older) patients admitted to the general surgery operating theatres at KCH in the calendar years 2007 - 2010. Kamuzu Central Hospital (KCH) is an 800 bed tertiary hospital in the capital city, Lilongwe. It serves as the referral center for the central region of Malawi, with a catchment population of 6 million persons. Diagnostic capabilities include a clinical laboratory for basic investigations (complete blood count, chemistry, microbiology) and a radiology department with plain and contrast radiography and ultrasonography facilities. At KCH, computed tomography has only recently been introduced and its availability is extremely limited. Mammography facilities are not available at this hospital. The Department of Surgery has three fully trained general surgeons, multiple clinical officers and a residency training program. The Malawi ministry of health has not adopted any formal cancer-screening program. There were no trained Oncologists in the country during the study; radiation therapy is not available, and chemotherapy is limited and subject to availability.

Data obtained from the operating theatre logs included patient demographics, indication for operative procedure, procedure performed, and operative intent. We further classified operative procedures based on operative intent (curative, palliative, or staging). Cancer diagnosis was based on clinical characteristics and histopathology, if available. Solid masses that were suspected to be clinically malignant in the absence of pathological diagnosis and were managed with palliative surgical procedures (for example, bypass, gastrostomy tube, tracheostomy) were included as cancer. Estimates of the number of fully trained long-term (commitment greater than 6 months) general surgeons in the central region of Malawi was obtained from the Malawi Medical Council. Both the Institutional Review Board of The University of North Carolina and the National Health Sciences Research Committee of Malawi approved this study.

## Results

During the study period, 7,076 surgical procedures were recorded in the operating theatre logs. Of these operations, 405 (6%) were cancer-related procedures for surgical cure, palliation or staging.

The most common cancers in women were breast cancer, colorectal cancer, gastric cancer, melanoma, and pancreatic cancer, while the most common cancers in men were prostate cancer, colorectal cancer, pancreatic cancer, gastric cancer, and esophageal cancer (Table 1). Breast cancer was seen exclusively in female patients. Colorectal cancer and gastric occurred with nearly equal frequency in males and females.

**Table 1:** Demographics of General Surgery Patients With Cancer

Site	n	%	Males (n)*	%	Females (n)*	%	Age	SD
Breast	90	22%	0	0%	90	100%	47.1	13.53
Colorectal	62	15%	32	52%	30	48%	45.86	17.87
Prostate	45	11%	45	100%	0	0%	70.55	10.57
Gastric	41	10%	27	66%	14	34%	52.06	16.54
Pancreatic	39	10%	19	49%	20	51%	54.51	12.67
Melanoma	26	6%	8	31%	18	69%	52.48	17.55
Esophagus	23	6%	14	61%	9	39%	49.19	15.80
SCC	13	3%	8	62%	5	38%	43.25	17.49
Penis	12	3%	12	100%	0	0%	54.67	15.85
Bladder	9	2%	4	44%	5	56%	31.75	19.94
Sarcoma	8	2%	6	75%	2	25%	53.5	16.12
Renal	8	2%	5	63%	3	38%	36.5	16.74
Skin NOS	5	1%	1	20%	4	80%	45.2	24.39
Liver	5	1%	4	80%	1	20%	34.00	9.8
KS	5	1%	0	0%	5	100%	57	3.46
Thyroid	4	1%	1	25%	3	75%	24.25	23.04
Other	4	1%	2	50%	2	50%	19.5	15.70
H&N	4	1%	4	100%	0	0%	50.75	29.20
Gynecologic	2	1%	0	0%	2	100%	22.5	3.54
Total	405	100%	192	49%	213	51%	50.34	17.92

**Figure 1:**
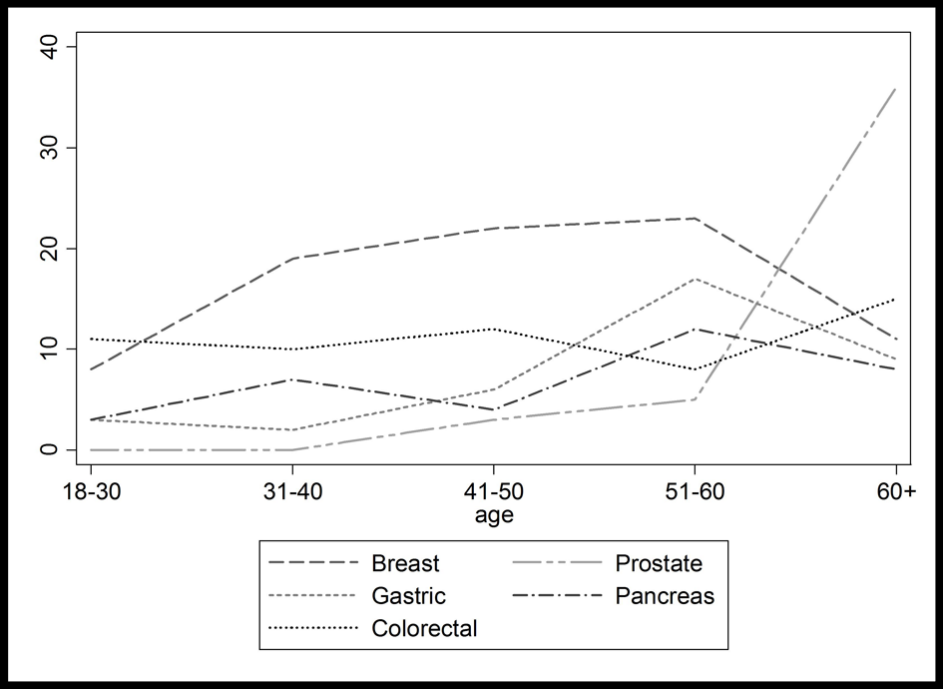
Most common cancer types by age.

The mean age of male and female patients in this study population was 52 years and 47 years, respectively. Colorectal cancer was least correlated with age and was operated as frequently in 18 - 30 year old patients as in 31 - 60 year old patients. Breast, pancreatic, and gastric cancers also occurred in young patients; with average ages of 47.1 years, 52.1 years, and 54.5 years respectively. Prostate cancer occurred in older patients, with a mean age of 70.6 years (Fig. 1).

**Figure 2:**
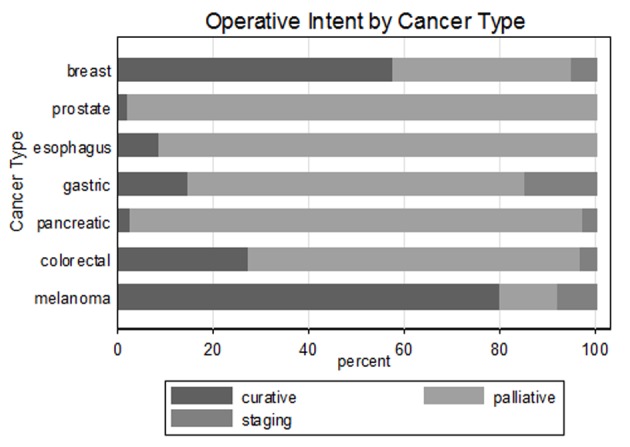
Operative intent by cancer type.

Operative procedures were categorized with respect to the operative intent: that is, curative, palliative, or staging (Fig. 2). Breast cancer and melanoma had the highest percentage of operations performed with curative intent (55% and 80%, respectively). Alternatively, prostate, gastric, pancreatic, and esophageal cancers were primarily treated with palliative procedures (in 98%, 71%, 94%, and 91% of cases, respectively). Two patients underwent skin grafting following palliative mastectomies, but no patient underwent reconstructive surgery following a curative excision.

## Discussion

Breast and colorectal cancers are the leading cancer diagnoses in our population and afflict both young and elderly adults with similar frequency. The majority of procedures were of palliative intent rather than curative or staging.

The preponderance of palliative operations performed in our population is remarkable. Prostate cancer was nearly exclusively treated with palliative bilateral sub-capsular orchiectomy; only one patient underwent a potentially curative prostatectomy. Gastric and pancreatic cancers were primarily treated with bypass or feeding jejunostomy. Likewise, esophageal cancers were palliated with feeding tube, tracheostomy, or endoscopic stenting. This is in contrast to the developed countries where surgical procedures for the most common cancers are of a curative intent including colorectal, breast and lung [7-9].

Multiple factors contribute to the paucity of curative surgical procedures in our population. Although screening mammography, colonoscopy and computed tomography are mainstays of cancer diagnosis and staging, none of these modalities is readily available at this institution. Radiotherapy is a scarce resource in sub-Saharan Africa which further exacerbates the problem of implementing curative strategies [10]. Furthermore, limited surgical capacity and access also hinders timely diagnosis and treatment.

Increasing surgical capacity is an important step to improving the care of cancer patients in Malawi. Several metrics for surveillance of surgical care in developing countries include the numbers of surgeons, anesthetists, theatres and surgical cases, in addition to postoperative mortality rates (day-of surgery mortality and in-hospital mortality) [11]. Kamuzu Central Hospital recently began a five-year surgical training program to address the shortage of surgeons in Malawi [12]. Additionally, KCH was historically unable to perform pathology consistently or in a timely fashion [13]. However as of mid 2012 through a partnership with UNC pathology is readily available at KCH with a turn-around time of less than one week [14, 15].

Our study population was limited to general surgery, and did not collect data on gynecologic or orthpaedic cancers. Cervical cancer disproportionately afflicts women in developing countries [16]. Additionally the data is hospital-based and does not include medical ward patients. Diagnostic procedures such as lymph node biopsy, cystoscopy, breast biopsy, and skin biopsy were not included if the diagnosis of cancer was not established. Lastly there is no outcome or follow-up data available though we intend to improve this gap in knowledge via the recently implemented cancer registry.

Our study revealed that most procedures were palliative, consistent with overall worse outcomes for cancer care observed in sub-Saharan Africa. The lack of adequate screening, staging, and therapeutic options (medical and surgical) only compounds the problem. Many cancers also presented at a young age further magnifying the years of life that are lost. Though KCH now has pathology services, a cancer registry and a surgical training program, the majority of surgical care unfortunately remains palliative. Further research should address other methods of increasing early cancer detection and treatment to address the increasing burden of disease from cancer in such populations.
